# Single-Nucleotide Polymorphisms Promote Dysregulation Activation by Essential Gene Mediated Bio-Molecular Interaction in Breast Cancer

**DOI:** 10.3389/fonc.2021.791943

**Published:** 2021-12-02

**Authors:** Xue Wang, Zihui Zhao, Xueqing Han, Yutong Zhang, Yitong Zhang, Fenglan Li, Hui Li

**Affiliations:** Department of Biochemistry and Molecular Biology, Harbin Medical University, Harbin, China

**Keywords:** breast cancer, differential expression, survival analysis, single-nucleotide polymorphisms, machine learning

## Abstract

**Background:**

Breast cancer (BRCA) is a malignant tumor with a high mortality rate and poor prognosis in patients. However, understanding the molecular mechanism of breast cancer is still a challenge.

**Materials and Methods:**

In this study, we constructed co-expression networks by weighted gene co-expression network analysis (WGCNA). Gene-expression profiles and clinical data were integrated to detect breast cancer survival modules and the leading genes related to prognostic risk. Finally, we introduced machine learning algorithms to build a predictive model aiming to discover potential key biomarkers.

**Results:**

A total of 42 prognostic modules for breast cancer were identified. The nomogram analysis showed that 42 modules had good risk assessment performance. Compared to clinical characteristics, the risk values carried by genes in these modules could be used to classify the high-risk and low-risk groups of patients. Further, we found that 16 genes with significant differential expressions and obvious bridging effects might be considered biological markers related to breast cancer. Single-nucleotide polymorphisms on the *CYP24A1* transcript induced RNA structural heterogeneity, which affects the molecular regulation of BRCA. In addition, we found for the first time that *ABHD11-AS1* was significantly highly expressed in breast cancer.

**Conclusion:**

We integrated clinical prognosis information, RNA sequencing data, and drug targets to construct a breast cancer–related risk module. Through bridging effect measurement and machine learning modeling, we evaluated the risk values of the genes in the modules and identified potential biomarkers for breast cancer. The protocol provides new insight into deciphering the molecular mechanism and theoretical basis of BRCA.

## Introduction

BRCA is a highly prevalent malignant tumor that presents serious threats to life and health around the world. Latest data show that the global incidence of breast cancer is increasing at a rate of 3.1% per year, and the rate of mortality from breast cancer remains high (1). Numerous studies have determined that BRCA is a heterogeneous disease whose development is linked to various environmental and genetic risk factors (2). However, the molecular mechanisms of breast cancer are still unclear, and further clarification of the molecular interaction and regulatory pathways, identification of key biological markers, and characterization of the genetic background of susceptibility factors are urgent so as to better elucidate the stage, prognosis, and risk features of this disease.

In recent years, with the continuous development of large-scale, high-throughput sequencing technologies, as well as the accumulated massive resources—which can be analyzed through a series of computational methods, artificial intelligence, and deep learning algorithms—a novel approach to the exploration of the molecular mechanism of tumorigenesis and tumor development has been realized. At present, breast cancer has been investigated in the fields of genomics (3), epigenetics (2, 4), metabolomics (5), and proteomics (6, 7). Integration of clinical prognostic information with whole genome sequencing data is an effective protocol to explore the molecular mechanism of breast cancer.

Based on the genomic expression information, module-based algorithm is one of the commonly used methods to explore the molecular mechanism of breast cancer by mining the global co-expression network modules and identifying intracellular molecular interactions (8, 9). For example, Niemira et al. identified key modules and genes in non–small-cell lung cancer through WGCNA. As a result, new hub genes were identified, including *CTLA4*, *MZB1*, *NIP7*, and *BUB1B* in adenocarcinoma as well as *GNG11* and *CCNB2* in squamous cell carcinoma (10). Yin et al. indicated that key genes were crucial bridge molecules for the interaction of intracellular biomolecules and play a predominant role in the coordination of co-expression networks because of their high connectivity; thus, hub genes might serve as vital biological marker or candidate drug target (11). However, a large number of hub genes were obtained in the above studies, and it is difficult to accurately focus on only the molecules with major effect factors in deciphering the essential regulation pathways. Aiming to explore the mechanism of the carcinogenesis and progression of cancer, the construction of a breast cancer risk-prediction model based on the effects of leading genes is extremely important (12).

In this study, WGCNA was used to identify co-expression network modules based on the RNA sequencing (RNA-seq) of BRCA. According to the hypergeometric test, we further screened modules enriched with differentially expressed genes. Next, by combining clinical information and taking advantage of survival analysis, a total of 42 breast cancer survival–related modules were identified. Finally, we introduced a machine learning algorithm to construct a prognostic risk model of breast cancer using the mined module information. The analysis of the expression of hub gene and single-nucleotide polymorphism (SNP) allosteric risk in the modules showed that 16 genes might be potential key biomarkers, as well as alternative drug targets. This study will likely help researchers to further comprehend the carcinogenesis and progression of breast cancer and could provide new insight into clinical treatment and drug research.

## Materials and Methods

### Data Processing

A breast cancer expression profile was downloaded using the HiSeq platform (Illumina, San Diego, CA, USA) from The Cancer Genome Atlas (TCGA) (13). A total of 96 tumor samples and their corresponding 96 adjacent normal samples in 1216 samples were obtained through sample matching which ensuring the results from same patients were reliable, and clinical information was also extracted for survival analysis. In addition, the remaining 974 samples after sample matching clinical details about the other breast cancer samples were adopted as a test set for internal validation. Genes with a read count of 0 in at least half of the samples were removed, and 30,089 genes were retained for further analysis. We converted the read count values of the genes into transcripts per kilobase of exon model per million mapped reads (TPM) (14) for co-expression network construction using a formula as follows:


$$TPM_{i}=\frac{\left(\frac{N_{i}}{L_{i}}\right)*1000000}{sum(\frac{N_{i}}{L_{i}}+\ldots +\frac{N_{m}}{L_{m}})}$$

where *N_i_* is the number of reads mapped to gene *i*, *L*_i_ is the sum of the exon lengths of gene *i*, and *m* is the total number of genes, respectively.

### Identification of Co-Expression Network Modules

To explore the co-expression modules, we constructed co-expression networks as undirected, weighted gene networks by WGCNA (9). The nodes indicated genes, and edges were determined by pairwise correlations between any two genes. The adjacency matrix was constructed to describe the correlation strength between genes. The value of adjacency matrix *a_ij_* was calculated as follows:


$${\text{a}}_{ij}=|cor(g_{i},g_{j}){|}^{\beta }$$

where *i* and *j* represented two different genes; *g_i_* and *g_j_* indicated their respective expression values (TPM); and β is the parameter representing the characteristics of scale-free network. In this study, the adjacency matrix met the scale-free topology criterion when the soft-threshold *β* equaled *5*.

Then, in order to identify co-expression network modules, a topological overlap matrix (TOM) was constructed based on the topological similarity between genes and hierarchical clustering. Using the standard R software program (R Foundation for Statistical Computing, Vienna, Austria) function *hclust*, we gathered the genes with high topological similarity and applied the dynamic branch cut methods to cut off different branches to obtain co-expression modules. The number of genes contained in each module was limited to at least 30.

### Analysis of Differentially Expressed Genes

The R package *DESeq2* was used to identify differentially expressed genes (DEGs) between BRCA tumor samples and normal samples. Genes with a count of less than 20 in the samples were filtered out, and genes with an adjusted *P*-value (Bonferroni, *p-adj*) of less than 0.01 and log2 |fold change (FC)| of at least 1 were considered to indicate significantly differential expression.

### Selection of Differentially Co-Expression Modules

In order to acquire differentially co-expressed modules (DCEMs), we conducted a hypergeometric test using the following equation:


$$P\;\text{value}=\Sigma_{i=m}^{M}\frac{\left(\underset{i}{M}\right)\left(\underset{n-i}{N-M}\right)}{\left(\underset{n}{N}\right)}=1-\Sigma_{i=0}^{m-1}\frac{\left(\underset{i}{M}\right)\left(\underset{n-i}{N-M}\right)}{\left(\underset{n}{N}\right)},$$

where *N* is the number of genes in the co-expression network, *M* is the number of genes in the co-expression modules, *n* is the number of DEGs, and *m* is the number of intersects of *M* and *n*. Modules with *P*-values of less than 0.05 were considered to be differentially co-expressed modules.

### Identification of BRCA Survival–Related Modules

A univariate Cox proportional hazards regression model (15) was used to analyze the association between the expression of genes and survival time by *coxph*. The risk score of a DCEM in patient *i* was calculated as follows:


$$risk\;score=\sum_{j=1}^{k}a_{j}*E{\left(\text{gen}{\text{e}}_{j}\right)}_{i}$$

where *α_j_* is the regression coefficients of gene *j* in Cox regression model, *k* is the number of genes in a candidate module, and *E (gene_j_)* is the TPM of gene *j*.

All of the tumor patients were divided into the following two groups based on the median of risk scores (MRS) of DCEMs: high risk (> MRS) and low risk (< MRS). Survival time was assessed at the Kaplan–Meier plotter (16), where results with a log-rank *P*-value of less than 0.05 were considered BRCA survival–related modules.

### Functional Enrichment Analysis

The R package *clusterProfiler* (17) was used to perform Gene Ontology (GO) and Kyoto Encyclopedia of Genes and Genomes (KEGG) pathway enrichment analyses for BRCA survival–related modules. GO functional annotations, including biological process (BP), cellular component (CC), and molecular function (MF), were obtained, which were considered statistically significant when the *P*-value was less than 0.05.

### Establishing the Risk Assessment Model

We integrated gene expression; risk scores; and clinical data, including age, histological type, tumor/lymph node metastasis (TNM stage), estrogen receptor (ER), progesterone receptor (PR), and human epidermal growth factor receptor 2 (HER2); constructing models for the one-, three-, and five-year survival probability prediction. Univariate analysis and hazard rate calculation were set up by the R package *rms*. Prediction model correction curves based on bootstrapping were applied to illustrate the uniformity between the practical outcomes and model prediction.

### Quantitative Real-Time Polymerase Chain Reaction (qRT-PCR)

The experimental BRCA cell line MCF-7 and normal human breast cell line MCF-10 were obtained from the biometrics cell bank of Wanlei. DMEM/F12 with 5% horse serum added was used for the culture of MCF-7 cells. All cells were cultured in a humidified environment consisting of 95% air and 5% CO_2_ at 37°C. Total RNA Extraction and qPCR Analysis RNase inhibitor (Beyotime Shanghai, Shanghai, China) and 10 µL of SYBR Master Mix (Solarbio, Beijing, China) were used to extract total RNA according to the protocol provided by the manufacturer (Solarbio, Beijing, China). qRT-PCR was conducted in triplicate. β-actin was used as an internal control, and the 2^−ΔΔCt^ values were normalized. The primer sequences for qPCR used in this study are shown in **Supplementary Table S1**.

## Results

### Exploring WGCNA

**Figure 1:**
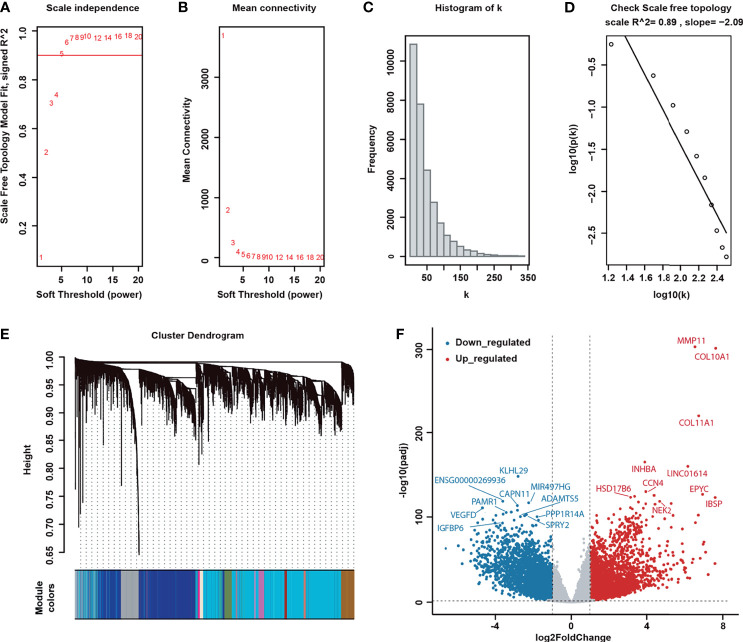
Determination of co-expression modules and differentially expressed genes in the weighted gene co-expression network analysis (WGCNA). **(A)** Scale-free index analysis of varying threshold powers (β). **(B)** The mean connectivity analysis of various soft-threshold powers. **(C)** The frequency of network connectivity (K). **(D)** Checking the scale-free topology when β is equal to 5. The x-axis represents the logarithm of whole network connectivity, and the y-axis shows the logarithm of the corresponding frequency distribution. The distribution follows an approximately straight line (R^2^ = 0.89), termed scale-free topology. **(E)** Modules mined by the WGCNA algorithm. **(F)** Differentially expressed genes of BRCA. An adjusted *P*-value (Bonferroni, *p-adj*) of less than 0.01 and log2 |fold change (FC)| of at least 1 were considered to suggest significantly differential expressions.

We constructed a weighted co-expression network based on 30,089 genes by WGCNA (see Materials and Methods section for details) Due to the threshold setting principle, when β was set to 5, the gene-interaction network attributed a scale-free network to present the optimal network connectivity state (R2 = 0.89; **Figures 1A–D**).

The genes with high topological similarity were collected by hierarchical clustering and a dynamic branch-cutting method to obtain the co-expression modules. Eventually, we identified 111 co-expression modules with sizes ranging from 32 to 3,156 genes (**Figure 1E**). Through differential expression analysis *via DESeq2*, we identified 7,629 DEGs, including 3,827 upregulated genes with log2 FC of at least 1 and 3,802 downregulated genes with log2 FC of −1 or less. In **Figure 1F**, the dark blue dots are downregulated genes, and the red dots are upregulated genes. GO function and KEGG annotation illustrated that DEGs potentially associated with cancer-related molecular regulation pathways, including the PI3K–Akt signaling pathway, Ras signaling pathway, JAK–STAT signaling pathway, and MAP kinase activity and negative regulation of cell adhesion (**Supplementary Table S2**).

### Identification of Breast Cancer Survival–Related Modules

Next, we further aimed to identify survival-associated modules in breast cancer based on the above differential expression analysis. After hypergeometric testing (*P* < 0.05), we retained 45 DCEMs with enrichment DEGs. Kaplan–Meier survival analysis and log-rank testing were conducted to evaluate the performance of prognosis. The modules with *P*-values of less than 0.05 were considered as cancer survival–related modules (see the Materials and Methods section for details). Ultimately, 42 breast cancer survival–related modules were detected (**Supplementary Table S3**). After DrugBank database retrieval, 35 of the 42 (88.33%) survival-related modules had at least one gene were targets that approved drugs by the United States Food and Drug Administration (FDA). The proportion of drug targets in survival-related modules (8.01%) was significantly larger than that in the total co-expression network (6.20%; Fisher’s exact test, *P* = 1.22 × 10^−9^) and in the co-expression modules (6.27%; Fisher’s exact test, *P* = 6.19 × 10^−9^). These results indicated that the genes in survival-related modules preferred to be considered with related targeted drugs.

**Figure 2:**
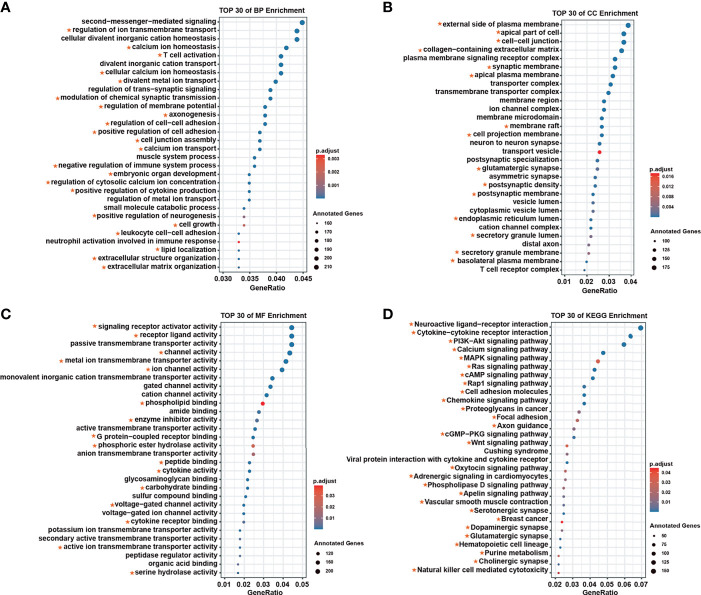
The top 30 annotations for survival-related modules. **(A)** Biological process enrichment. **(B)** Molecular function enrichment. **(C)** Cellular component enrichment. **(D)** Kyoto Encyclopedia of Genes and Genomes enrichment. The red star represents the confirmed function associated with breast cancer in the Comparative Toxicogenomics Database.

We analyzed the biological functions and molecular regulatory pathways of the screened breast cancer survival modules in detail, finding the top 30 significantly enriched GO terms and KEGG pathways, which showed these modules were mainly involved in immune responses (**Figure 2**). For example, neutrophil activation is involved in the immune response, regulation of T-cell activation, cell growth and T-cell differentiation, which is related to GO terms. Based on pathway annotation, breast cancer–related modules were significantly related to drug-related processes, such as the PI3K–Akt signaling pathway, MAPK signaling pathway, and breast cancer and drug metabolism cytochrome P450 (**Supplementary Table S4**). Known breast cancer–related GO terms and KEGG pathways were collected from the Comparative Toxicogenomics Database (CTD) (18). Notably, there was considerable overlap between the functions annotated with the survival-related modules and the breast cancer–related functions recorded in the CTD. The consistency of enrichment results of BP, CC, MF, and KEGG with CTD totaled 63.9%, 53.9%, 57.5%, and 77.8%, respectively (**Supplementary Table S4**).

**Figure 3:**
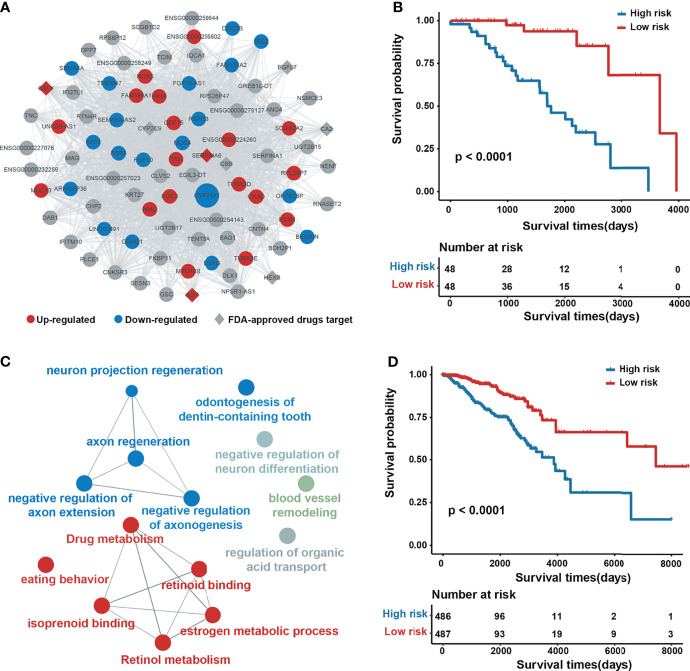
Network architecture and prognostic analysis of the salmon2 module. **(A)** Network plot of the salmon2 module. The upregulated, downregulated, and non–differentially expressed genes are colored with red, blue, and gray, respectively. The rhombus represents the drug target. The size of a node represents the degree of connectivity in the network. **(B)** Kaplan–Meier analysis for 96 patients with high-risk or low-risk scores. *P*-values were calculated using the two-sided log-rank test. **(C)** Gene ontology enrichment analysis of the genes in the salmon2 module. **(D)** Kaplan–Meier analysis for 974 independent verification patients.

Take the salmon2 module as an example (**Figure 3A**), which contains 87 genes, of which 17 are upregulated, 21 are downregulated, 6 can be targeted by FDA-approved drugs, and 58 genes are associated with BRCA in CTD. Using expression information contained in the genes of salmon2, we conducted Kaplan–Meier survival analysis on 96 breast cancer samples. Samples were divided into a low-risk group (n = 48 patients) and high-risk group (n = 48 patients) based on the median value of risk scores (see the Materials and Methods section for details). The results showed that this module covers a favorable classification and prognostic function, with a log-rank test *P*-value of less than 1.0 × 10^−4^ (**Figure 3B**). The functional annotation of salmon2 was enriched by metabolism and regulation-related paths, such as the estrogen metabolic process and drug metabolism (**Figure 3C**). To demonstrate the potential prognosis effect of the salmon2 module, we collected an independent verification set with additional 974 cancer samples. The subsequent Kaplan–Meier survival analysis was performed by utilizing the identical principle and revealed that the genes in salmon2 exhibited a dominant prognostic capability, with a log-rank test *P*-value of less than 0.0001 (**Figure 3D**). The findings indicated that the survival-related modules detected by our pipeline might serve as potential prognostic biomarkers of breast cancer.

### Selection of Biomarkers for Breast Cancer

**Figure 4:**
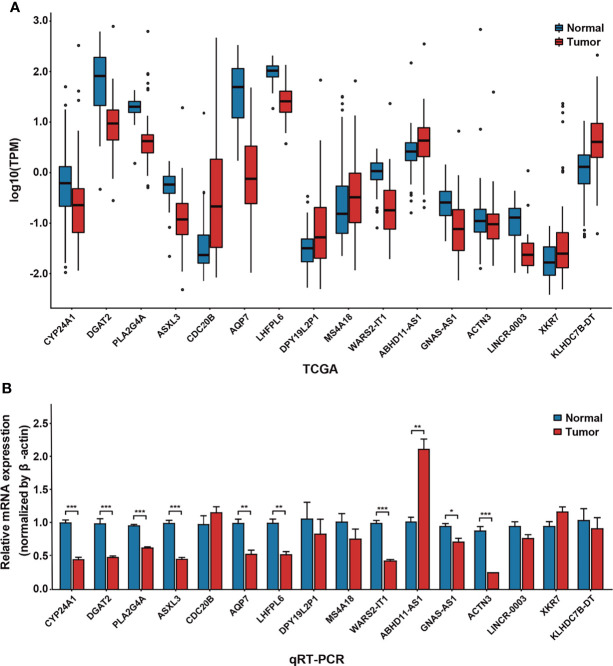
Verification of differential expression of hub genes by quantitative real-time polymerase chain reaction (qRT-PCR). **(A)** Expression of 16 hub genes in 96 cancer samples and 96 adjacent cancer samples, obtained from the Cancer Genome Atlas. **(B)** Expression of 16 hub genes in the MCF-7 cell line and normal breast cells by qRT-PCR. *P < 0.05; **P < 0.01; ***P < 0.001.

Hub nodes with a high degree of connection in the modules may play a crucial role in biological regulation processes. Subsequently, we screened 42 hub genes with bridging roles in the survival-related modules as candidate biomarkers, including 12 downregulated (log2FC < −1, *p-adj* < 0.01) and eight upregulated (log2FC > 1, *p-adj* < 0.01) genes. Among these, 28 were confirmed to be related to breast cancer according to CTD annotation and literature mining (**Supplementary Table S5**). After removing pseudogenes and other genes with no corresponding names, we selected 16 genes for qRT-PCR expression verification. The expression level of most of the genes (13, 81.25%) was consistent with the results of data mining (**Figure 4**). *ABHD11-AS1* was highly expressed in breast cancer samples, which means that the value of expression was 2.13 and the *P*-value was 0.003. These findings proved that the discovered hub genes are credible biomarkers, which may contribute to bridging molecular interactions.

**Figure 5:**
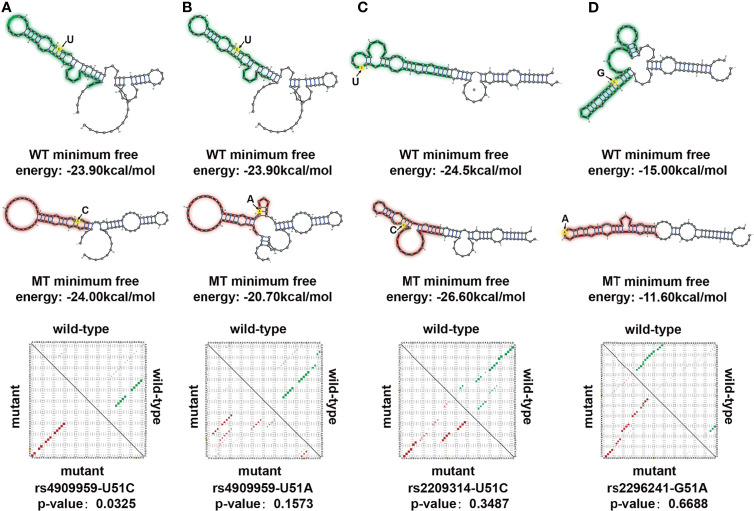
Allosteric effect analysis of *CYP24A1* induced by single-nucleotide polymorphisms (SNPs). **(A)** RNA structure disturbance deduced by the rs4909959 UC allele. Changes in structure were identified by RNAsnp. Green represents wild type (WT), red represents mutant type (MT). SNP position is colored in yellow. Minimum free energy describes the stability of the RNA structure. The base pair probability of the local RNA secondary structure is shown in the dot plot, with the upper triangle representing wild type (green) and the lower triangle representing mutant type (red). **(B)** RNA structure disturbance deduced by the rs4909959 UA allele. **(C)** RNA structure disturbance deduced by the rs2209314 UC allele. **(D)** RNA structure disturbance deduced by the rs2296241 GA allele.

Interestingly, the hub gene *CYP24A1* in the salmon2 module mentioned above was downregulated in breast cancer samples, consistent with numerous previous reports (19). CYP24A1 was an essential gene in regulation of vitamin D. It had been reported to play an important role in enhancing immune activity and inhibiting tumorigenesis (20, 21). In order to further decipher the molecular mechanism of *CYP24A1*, we identified three known breast cancer–related SNPs (rs4909959, rs2209314, rs22762941) according to the SNP4Disease database (22, 23). Flanking sequences of SNPs (50 bp upstream and downstream of mutant alleles) were also obtained using the dbSNP database (24). Next, RNAsnp (25) was used to compare the RNA secondary structural changes between wild- and mutant-type transcripts. The rs4909959 U51C allele (*P* = 0.0325) substitution resulted in a minimum free energy (MFE) value range of −23.90 to −24.00 kcal/mol and U51A allele (*P* = 0.1573) substitution resulted in an MFE value range of −23.90 to −20.70 kcal/mol. Green regions in **Figure 5** represent wild-type and red represents mutant-type transcripts, respectively. We could observe the obvious structural changes in local regions induced by rs4909959, especially at the U51C allele. The number of internal loop structures changed, with bulge loops disappearing. Also, the number of bases contained in hairpin loops increased significantly (**Figures 5A, B**). In addition, the base-pairing probability was disturbed visibly from the square dot plot of **Figure 5**. The upper triangle for wild-type (green) and the lower triangle for mutant-type (red) transcripts indicate that there was a significant allosteric effect on the folding morphology of wild-type and mutant RNA transcripts, respectively. The other two substitutions in SNP, the rs2209314 U51C allele (*P* = 0.3487) and rs2296241 G51A allele (*P* = 0.6688), contributed to an MFE decrease in the range of −24.50 to −26.60 kcal/mol and −15.00 to −11.60 kcal/mol. **Figure 5C** exhibited a change from hairpin loops to stem loops, while the change from stem loops to hairpin loops was shown in **Figure 5D**. Structural variants can lead to phenotypic variation or disease in several ways, which can indirectly change gene expression through location effect. In addition to these potentially pathogenic changes in gene expression, the presence of structural variations may also lead to further, potentially harmful structural changes (26). So the dominant structural heterogeneity demonstrated that SNPs induce changes in the RNA folding, triggering a disturbance in the ability of molecular interactions, thereby affecting the network bridging and combining effect with breast cancer–related molecular regulatory pathways, ultimately leading to the occurrence and progression of the disease. We speculated that known BRCA-associated SNPs might influence the structure of *CYP24A1*, which might be a significant molecular basis of its potential biomarker.

In addition, *ABHD11-AS1* was a key gene with a significantly high expression status in the breast cancer survival–related modules, which was identified in a novel fashion by our study. In previous studies, *ABHD11-AS1* has been confirmed as a prognostic marker for lung, ovarian, thyroid, and pancreatic cancers and so on, but there was still no evidence to support a link to breast cancer prognostic. Therefore, we thought it worth digging deeper into its role in breast cancer.

### Construction of the Prognostic Risk Model of Modules

We next established a risk prediction model based on univariate and multivariate Cox regression analyses to evaluate the prognostic performance of the detected modules by integrating genetic effects and clinical characteristics of BRCA survival modules. The clinical features included age, histological type, TNM staging, ER, PR, and HER2 (**Table 1**). We referred to the risk ratios of 42 modules and the corresponding significant *P*-values, along with the importance score of each clinical feature in the model.

**Table 1:** Clinical characteristics of 96 samples.

Features	Total	Alive	Dead
**Number**	96	66	30
**Age (median (IQR))**	56.00 (45.00–63.50)	56.00 (43.00–63.00)	60.00 (48.50–75.75)
**Histological_type (%)**			
Infiltrating Ductal Carcinoma	76 (80.0)	52 (80.0)	24 (80.0)
Infiltrating Lobular Carcinoma	5 (5.3)	4 (6.2)	1 (3.3)
Mixed histology	10 (10.5)	8 (12.3)	2 (6.7)
Other	4 (4.2)	1 (1.5)	3 (10.0)
**Stage (%)**			
Stage I	16 (16.8)	10 (15.4)	6 (20.0)
Stage II	58 (61.1)	40 (61.5)	18 (60.0)
Stage III or higher	21 (22.1)	15 (23.1)	6 (20.0)
**ER (%)**			
Negative	16 (16.8)	11 (16.9)	5 (16.7)
Not Evaluated	12 (12.6)	7 (10.8)	
Positive	67 (70.5)	47 (72.3)	20 (66.7)
**PR (%)**			
Negative	25 (26.3)	17 (26.2)	8 (26.7)
Not evaluated	11 (11.6)	7 (10.8)	4 (13.3)
Positive	59 (62.1)	41 (63.1)	18 (60.0)
**HER2 (%)**			
Negative	67 (70.5)	52 (80.0)	15 (50.0)
Not evaluated	6 (6.3)	0 (0.0)	6 (20.0)
Positive	22 (23.2)	13 (20.0)	9 (30.0)

**Figure 6:**
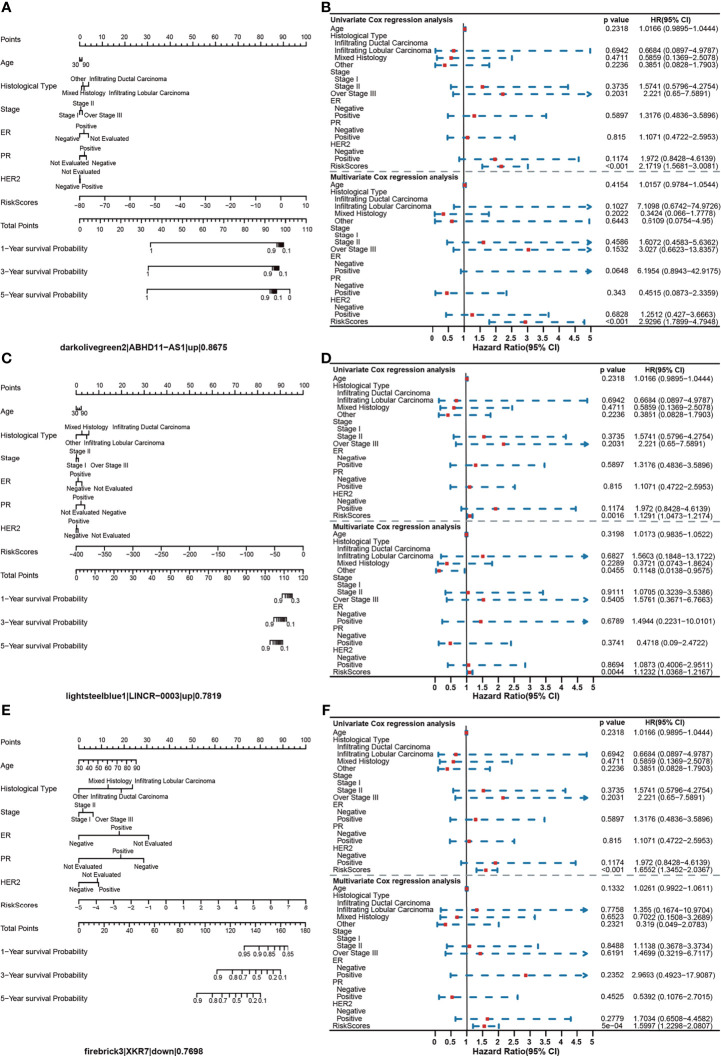
Assessment of the prognostic risk model for BRCA. **(A)** Nomogram model of the darkollvereen2 module, with the leading gene being *ABHD11-AS1*. **(B)** Univariate and multivariate regression analyses of the darkollvereen2 module. **(C)** Nomogram model of the lightsteelblue1 module, with the leading gene being *LINCR-0003*. **(D)** Univariate and multivariate regression analyses of the lightsteelblue1 module. **(E)** Nomogram model of the firebrick3 module, with the leading gene being *XKR7*. **(F)** Univariate and multivariate regression analyses of the firebrick3 module.

We found that all of the identified survival-related modules have a potential ability for BRCA risk assessment. The average value of C-index was 0.793. The lowest C-index of the dark red module was still 0.7123, which was above the experience threshold of 0.70 (**Supplementary Table S6**). Interestingly, we found a consistent phenomenon by the constructed nomogram models. In the risk score assessment of the nomogram, a model with a lower percentage of clinical features and a higher proportion of the gene risk value maintained a higher C-index. Furthermore, in univariate and multivariate regression analyses of all 42 modules, no remarkable risk scores associated with clinical characteristics were found, but a significant risk score for gene sets in each module (*P* < 0.001) was observed. In addition, the risk ratio of each feature in the nomogram chart can be broadly divided into three categories. In category 1, the risk scores of all clinical features were low. However, it is clear that there was one gene or several genes showing a main effect in the evaluation of the one-, three-, and five-year prognostic risk of BRCA (**Figure 6A**). In the darkolivegreen2 module, including the hub gene of ABHD*11-AS1*, the Hazard ratio (HR) was 2.1719 [95% confidence interval (CI), 1.5681–3.0081; *P* < 0.001)] by univariate Cox regression analysis and 2.9296 (95% CI, 1.7899–4.7948, *P* < 0.001) by multivariate Cox regression analysis (**Figure 6B**). The findings illustrated that the prognostic module with *ABHD11-AS1*, in combination with other clinical indicators, such as ER, PR, HER2, and TNM, have high accuracy and sensitivity for breast cancer risk-stratification (*C-index* = 0.868). In addition, the nomogram showed that up to 16.1% of the risk score was derived from the expression value of the genes in the module rather than clinical features. Among gene risk scores, ABHD11-AS1 has the highest risk score with 23.847. So it played a completely dominant role in the module, which further indicated the possibility and necessity of ABHD11-AS1 as a breast cancer–related risk target or biomarker. The greater the proportion of risk scores in the prediction model, the greater the consistency index of the model, indicating that risk scores could be better at predicting the prognosis of breast cancer, as shown in **Supplementary Figure 1**.

Category 2 was similar to category 1. The risk score of clinical characteristics in the nomogram was low, but the genetic risk score was relatively high and balanced (**Figure 6C**). Taking lightsteelblue1 module as an example, the HR was 1.1291 (95% CI, 1.0473–1.2174; *P* = 0.002) in the univariate Cox regression analysis and 1.1232 (95% CI, 1.0368–1.2167; *P* = 0.004) in the multivariate Cox regression analysis (**Figure 6D**). The C-index of the module was 0.7820, with the hub gene being *LINCR-003*. Category 3 models were slightly different; here, the effect sizes of clinical factors and genetic risk values were comparative (**Figure 6E**). The Firebrick3 module is representative of this type of module, where the HR was 1.6552 (95% CI, 1.3452–2.0367; *P* < 0.001) in the univariate Cox regression analysis and 1.5997 (95% CI, 1.2298–2.0807; *P* < 0.001) in the multivariate Cox regression analysis, respectively (**Figure 6F**). The C-index of the module was 0.7699, with the hub gene being *XKR7*. Overall, our findings indicated that the gene risk score of BRCA survival–related modules could be an independent feature to predict breast cancer prognosis.

## Discussion

In this study, we constructed co-expression network modules by WGCNA and identified biomarkers related to breast cancer prognosis by combining clinical features and RNA-seq data. The functional annotation of survival-related modules indicated that these modules were mainly involved in some immune responses, cancer pathways, and the metabolism of certain drugs. By analyzing the function and molecular mechanism of leading genes, we found that 16 key biomarkers of breast cancer might be related to prognosis and molecular diagnostics, including *CYP24A1* and *ABHD11-AS1*. Finally, we established a risk-prediction model using a machine-learning algorithm. Using univariate and multivariate regression analyses, we found that the expression risk carried by a gene can well predict the prognosis of breast cancer.

This study confirmed that the single nucleotide change of *CYP24A1* could induce the mutation sequence to change the folded state of the spatial structure. This structural heterogeneity might be the potential mechanism that caused *CYP24A1* to be significantly downregulated in breast cancer samples and participated in the specific molecular function of breast cancer. Therefore, we propose a hypothesis that SNP changes can cause RNA secondary structure changes, affecting gene expression and leading to the occurrence of diseases. Certainly, this hypothesis still needs to be validated by experiments in further studies.

Interestingly, evidence has demonstrated that *ABHD11-AS1* is closely correlated with an unfavorable prognosis of patients with non–small-cell lung cancer (27), bladder cancer (28, 29), ovarian cancer (30), thyroid cancer (31, 32), and other cancers, but *ABHD11-AS1* was first confirmed to have an association with breast cancer prognosis in this study.

Our analysis only took advantage of RNA-seq data, but a large number of studies have shown that microRNAs, lncRNAs, and epigenetic modifications was available for screening prognostic markers in cancer; thus, we can further integrate multiple omics data to dig out factors related to the prognosis of breast cancer. This will be conducive to a more comprehensive exploration of the factors related to the prognosis of breast cancer, a deeper understanding of the pathogenesis of breast cancer, and the provision of new ideas for the treatment of cancer and new targets for drug development.

In summary, we identified the modules related to breast survival in combination with expression data and clinical information and verified the results from different perspectives, such as functional enrichment, targeted drug enrichment, and risk model construction, indicating that the key genes in these modules can be used as biomarkers for breast cancer prognosis.

## Data Availability Statement

The original contributions presented in the study are included in the article/**Supplementary Material**. Further inquiries can be directed to the corresponding author.

## Author Contributions

XW, FL, and HL conceived and designed the study. ZZ and XH download and collated data. YuZ and YiZ performed the statistical analysis. XW and ZZ performed the data analysis. XW wrote the manuscript. HL revised the manuscript. All authors listed have made a substantial, direct and intellectual contribution to the work, and approved it for publication.

## Funding

This work was supported by the National Natural Science Foundation of China (Nos.81472929 and 81172616) to HL.

## Conflict of Interest

The authors declare that the research was conducted in the absence of any commercial or financial relationships that could be construed as a potential conflict of interest.

## Publisher’s Note

All claims expressed in this article are solely those of the authors and do not necessarily represent those of their affiliated organizations, or those of the publisher, the editors and the reviewers. Any product that may be evaluated in this article, or claim that may be made by its manufacturer, is not guaranteed or endorsed by the publisher.

## Supplementary Material

Supplementary Figure 1 Correlation analysis featuring carryover risk and model accuracy. **(A)** Scatterplot between C-index and risk effects of clinical characteristics. **(B)** Scatterplot between C-index and risk effects of genes.
