# Supplementary figures and images for: Single-Nucleotide Polymorphisms Promote Dysregulation Activation by Essential Gene Mediated Bio-Molecular Interaction in Breast Cancer
Source: Front Oncol. 2021 Dec 2;11:791943. doi: 10.3389/fonc.2021.791943 (PMC8674201; doi:10.3389/fonc.2021.791943)

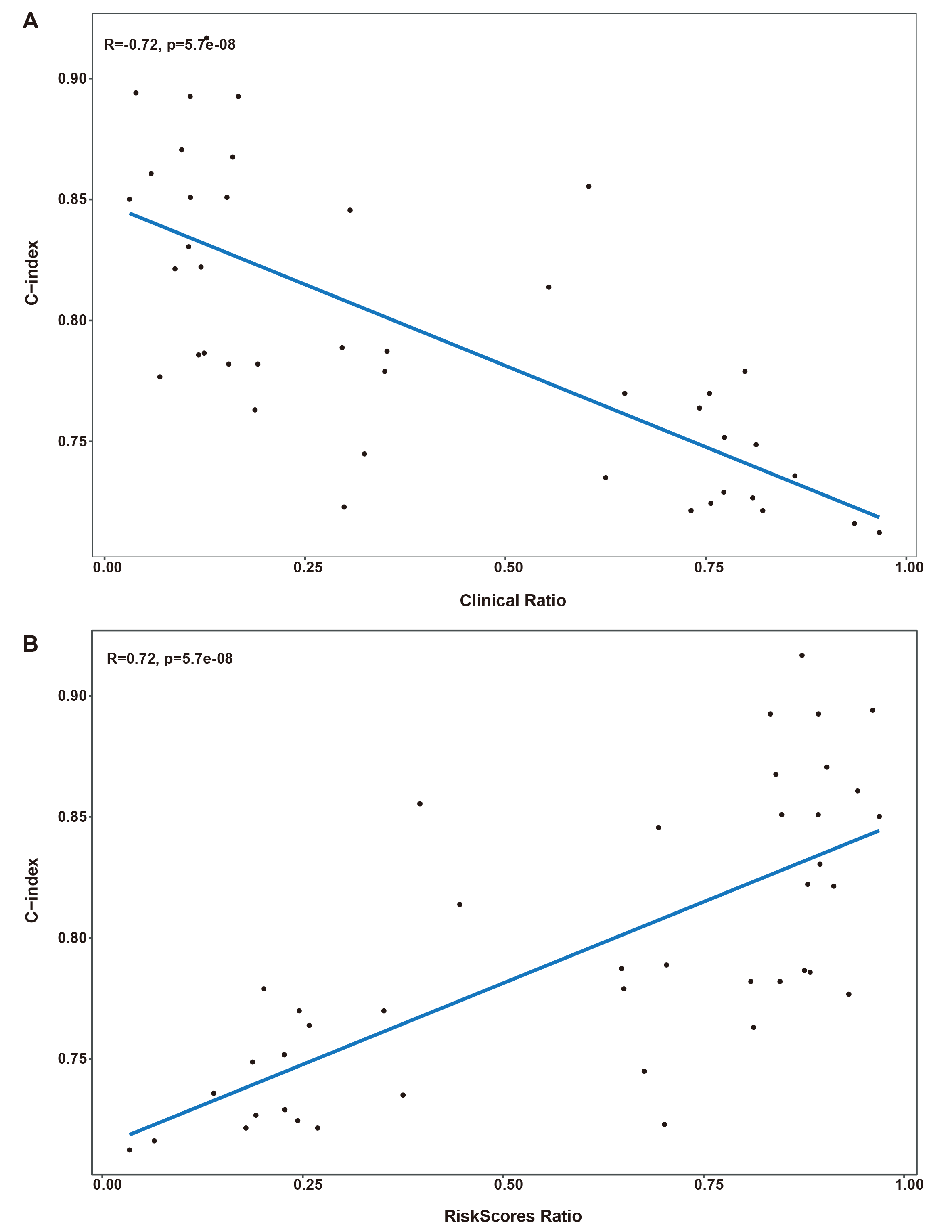

Supplement: Supplementary Figure 1 — Correlation analysis featuring carryover risk and model accuracy. (A) Scatterplot between C-index and risk effects of clinical characteristics. (B) Scatterplot between C-index and risk effects of genes. [file Image_1.tif]
